# Evaluation of Testicular Nonseminomatous Germ Cell Tumor Using Contrast-Enhanced Ultrasound

**DOI:** 10.1155/crra/6614645

**Published:** 2025-05-13

**Authors:** Vivek Gupta, Deepak Shah, Karan Gatiya, Shishira Shetty

**Affiliations:** ^1^Department of Radiology, Orange Clinic, Tarapur, Maharashtra, India; ^2^Department of Surgery, Palghar Nursing Home, Palghar, Maharashtra, India; ^3^Department of Pediatric Surgery, Orange Clinic, Tarapur, Maharashtra, India

**Keywords:** case report, CEUS, contrast ultrasound, perflutren microbubbles, testicular neoplasm

## Abstract

Ultrasound is the first-line and established imaging modality for the diagnosis of testicular masses and neoplasms, with high sensitivity. Conventional ultrasound can very reliably detect presence of an intratesticular mass although it may not specifically characterize them as benign or malignant and neoplastic or nonneoplastic or classify the type or subtype of neoplasm in every case. Contrast-enhanced ultrasound (CEUS) is a technique in which injected intravenous microbubbles can supplement the characterization of focal testicular masses through observation of real-time perfusion of the testis and the target mass. Testicular masses have been documented to demonstrate unique enhancing patterns on CEUS. We report a unique case involving a young male presenting with a palpable testicular mass in which CEUS aided in the diagnosis of testicular germ cell tumor. The diagnosis was subsequently confirmed by histopathological examination after surgery. This case report highlights the utility of CEUS as a useful complementary adjunct in diagnosing and characterizing nonseminomatous germ cell tumors of the testes with a review of the literature.

## 1. Introduction

With the increasing acceptance of contrast-enhanced ultrasound (CEUS) in diagnostic imaging, its applications have been extended and explored in a myriad of specialties, including ultrasound of testicular tumors, as highlighted in this study [1]. Although testicular tumors are rare, accounting for about just 1% of malignant tumors encompassing the whole body, they happen to be the commonest solid tumors in young men in the 20–40-year age bracket, all of which are malignant unless proven otherwise [2]. B-mode ultrasound is reputed to be the first-line imaging modality of choice in testicular pathologies and has a very high sensitivity in picking up focal testicular-occupied lesions [3].

Conventional ultrasound, however, may not always be able to determine the nature of lesions and distinguish malignant from benign lesions. The B-mode ultrasound appearance of testicular pathology can be nonspecific in some cases. The inclusion of contrast studies with conventional ultrasound is useful in such cases and increases the specificity of ultrasound in attaining lesion diagnosis. Ultrasound contrast agents like perflutren stay entirely within the lumen of blood vessels and do not perfuse to the extracellular space. Therefore, they are purely intravascular agents [4]. Certain contrast-enhancing patterns of various testicular pathologies on ultrasound have been observed and described, thereby complimenting in clinching diagnosis of the intratesticular pathology. Contrast ultrasound is increasingly becoming more accepted and familiar with radiologists and sonographers. This paper showcases such a case of intratesticular neoplasm, specifically a nonseminomatous germ cell tumor (NSGCT), in which ultrasound contrast was used as an adjunct to conventional ultrasound and helped classify the lesion. This study also highlights the convenience and ease with which ultrasound contrast agents can be applied in the same setting as conventional ultrasound and encourages the application of CEUS in systems beyond its traditional use like for hepatic and renal lesions.

## 2. Patient Information

A young male patient in his early twenties of Indian ethnicity presented to surgical OPD with gradually expanding painless left scrotal swelling. He was afebrile. No significant medical or family history was noted. There was no relevant psychosocial history or genetic information.

## 3. Clinical Findings

The swelling was firm on palpation and was nontender. There were no signs of inflammation. No lymph nodes were palpable. He was otherwise fit in the rest of his general physical examination. An ultrasound of the scrotum was requested.

## 4. Diagnostic Assessment

Scrotal ultrasound examination was performed on a Philips Epiq Elite ultrasound machine and revealed an enlarged left testis due to a 50-mm intratesticular mass.

**Figure 1:**
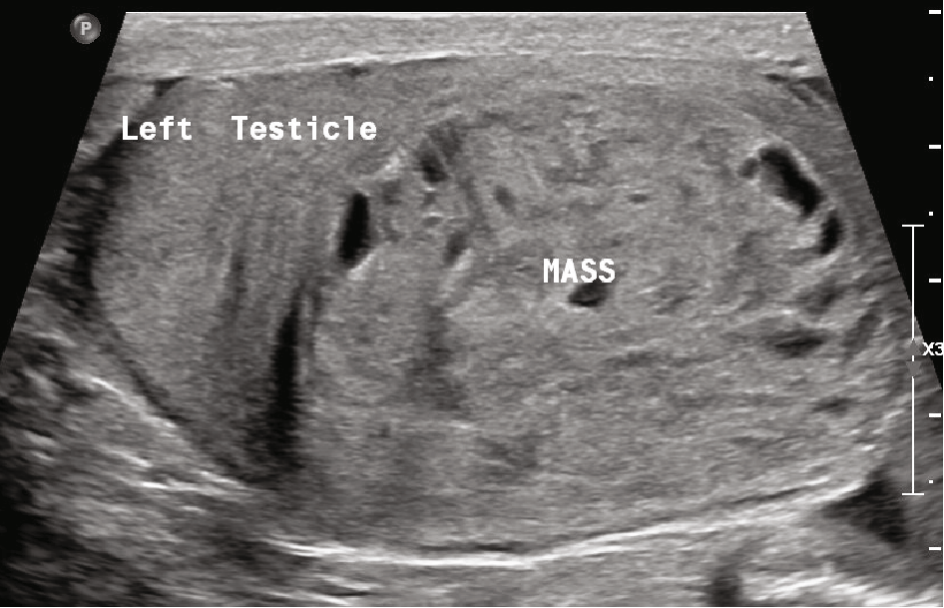
B-mode ultrasound reveals a well-circumscribed intratesticular mass lesion involving the left testicular parenchyma in the inferior aspect. The lesion is heterogeneous and predominantly solid with several small cystic spaces.

**Figure 2:**
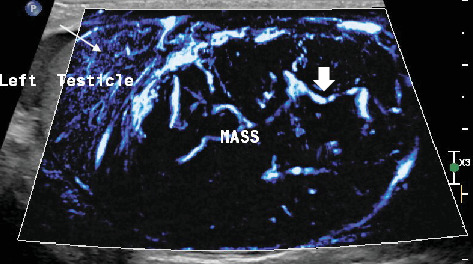
Marked intralesional vascularity and hyperemia on microflow imaging (MFI), a form of power Doppler imaging, in which vessels are seen as bright lines (thin long arrow). The image also depicts the typical curved and haphazard (short thick arrow) course of the vessels.

This mass within the left testis was ellipsoidal in shape, heterogeneous, and encapsulated; had lobulated margins; and was well circumscribed in the inferior part of the parenchyma (Figure 1). The lesion was solid with innumerable small cystic spaces randomly scattered throughout it corresponding to a “honeycomb” pattern. This lesion was hypervascular on color Doppler (Figure 2). No calcifications were identified. The presumption from conventional ultrasound was most likely a neoplasm. Differentials like hematoma or granulomatous lesions could not be ruled out. The surgeon wanted a more definitive diagnosis before proceeding to operate.

**Figure 3:**
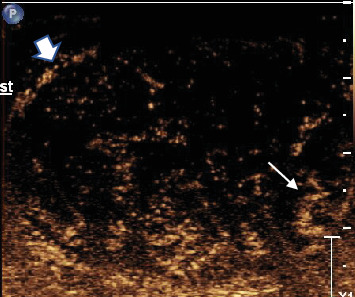
Contrast-enhanced ultrasound image of the lesion using perflutren microbubbles in the late arterial phase. The bubbles within intralesional vessels are bright echoes, whereas avascular areas remain dark or anechoic and represent cystic spaces or necrotic areas. The typical enhancing “twisted vessels” (thin long arrow) are identified within the tumor. The peripheral rim enhancement (short thick arrow) is also appreciated. Early wash-in and early wash-out of contrast were observed.

To further characterize the lesion, CEUS was performed using the agent Definity (perflutren microbubbles). Approximately 0.4 mL of this contrast was injected intravenously. The lesion demonstrated early wash-in and early wash-out which indicates malignancy rather than benign etiology. The lesion was heterogeneously hyperenhancing compared with normal testicular parenchyma with an arborized, haphazard, and “crisscross” pattern of vessels interspersed with multiple low or nonenhancing areas representing cystic or necrotic areas. Most intralesional vessels had curved or “twisted vessel” appearance (Figure 3).

Based on the B-mode characteristics combined with contrast-enhancing pattern, the lesion was reported as NSGCT with added confidence.

Subsequently, serum tumor marker levels were evaluated, which revealed elevated AFP, beta-HCG, and LDH levels. No metastases were detected. Other hematological tests, including CBC, were normal.

## 5. Therapeutic Intervention, Follow-Up, and Outcome

**Figure 4:**
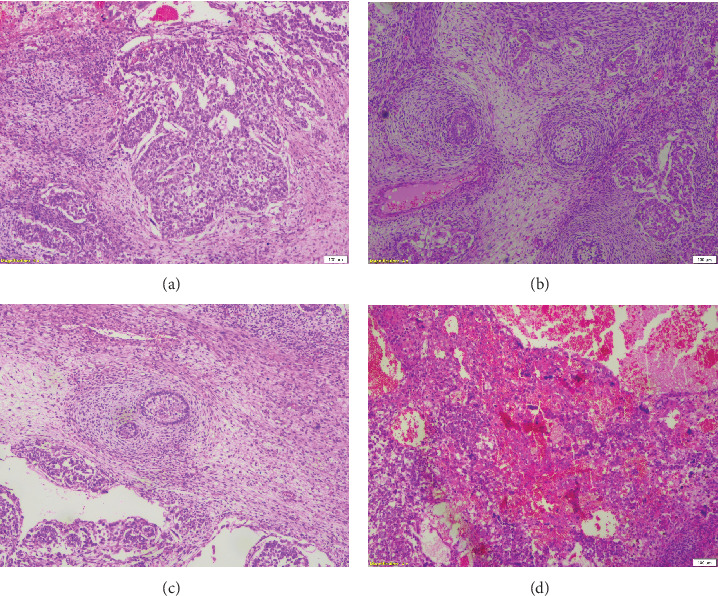
(a–d) Photomicrography of histopathological evaluation of the testicular mass. The tumor comprised 50% embryonal carcinoma (a), 20% yolk sac tumor (b), 20% mature teratoma (c), and 10% choriocarcinoma (d). This appearance confirmed a nonseminomatous germ cell tumor of mixed cell type. Pathological staging was pT3 (pTNM, AJCC eighth edition). Credit for histological images: Dr. Mrinal Kishor Mallya, MD, DNB, consultant histopathologist, P.D. Hinduja National Hospital, Mumbai, India.

Complete left orchidectomy was performed by the surgeon. No external spread of the tumor or abnormal lymph nodes were identified at the time of surgery. Histopathological examination revealed a NSGCT of mixed cell type (Figure 4). The patient subsequently underwent chemotherapy. He is now in remission and is reported to be doing well at a 2-year follow-up after surgery.

## 6. Discussion

CEUS has emerged as an encouraging novel modality, particularly as a problem-solving tool in diagnostic imaging. It has been widely accepted in the evaluation of hepatic and renal lesions; however, limited usage and data exist regarding the evaluation of intrascrotal lesions by CEUS.

In CEUS, a contrast agent is injected either intravenously or intracavitary and dynamically assessed on real-time ultrasound using a specialized contrast imaging mode. Second-generation contrast agents are currently in clinical use and are composed of microbubbles of an inert gas surrounded by a stabilizing shell. The contrast used in this study was Definity (Lantheus, United States), which is perflutren gas in a lipid shell. In addition to Definity, other contrast agents like Sonovue and Usphere are also commonly used ultrasound contrast agents. These ultrasound contrast agents remain purely intravascular, and unlike CT contrast, they do not extend to the extracellular space. They are approximately the size of erythrocytes and are tiny enough to permeate capillaries but large enough not to pass through the endothelium to the extracellular space. These nonionizing microbubble agents have a very low incidence of adverse reactions [4] and are not nephrotoxic. The incidence of life-threatening anaphylactic reactions in abdominal studies was 0.001% and with no deaths occurring in a series of > 23,000 patients [5].

Broadly, testicular neoplasms are classified as germ cell and nongerm cell types, of which the majority are germ cell type. Of these germ cell tumors, approximately 95% are malignant, with the most common subtype being seminomas. The prognosis is poor once metastasis occurs [6]. NSGCTs are classified into four major subtypes: embryonal carcinoma, yolk sac carcinoma, choriocarcinoma, and teratoma. A tumor comprising a combination of these subtypes is classified as a mixed cell germ cell tumor.

Conventional grayscale ultrasound detects testicular occupied lesions with high sensitivity and can aid somewhat in characterizing the lesions; however, the specificity in classifying these lesions may be low. Color Doppler can detect intralesional vascularity; however, it is usually limited to detect 1–2 mm blood vessels at most but is miserable in identifying and demonstrating those smaller than them [7]. Furthermore, it is observed that nonneoplastic lesions are devoid of significant vascularity in the majority compared with neoplastic lesions, which generally show neovascularity and marked small vessel proliferation [8]. Therefore, differentiating neoplastic with nonneoplastic lesions may often be difficult on conventional ultrasound but easier to classify when in adjunct with CEUS. Lesions determined to be indeterminate on conventional ultrasound often result in unnecessary surgeries and orchiectomy.

Contrast ultrasound is a novel technology that can significantly improve the display of low-speed blood flow and evaluate the blood perfusion characteristics of organs and lesions [9]. It can show the supplying blood vessels and the pattern of neovascularity, characterize the blood supply, and determine the nature of masses [10, 11].

One study observed fast-in and fast-out enhancement in seminomas and germ cell tumors, although fast-in with slow-out enhancement was also documented in a seminoma [9]. Heterogeneous enhancement was observed in all malignancies at the peak time.

Another study observed rapid enhancement in seminomas with loss of the normal linear vascular pattern [6]. Even with rapid washout, persistence of the abnormal “crossing” vessels was documented. In NSGCTs, as in this study, abnormal vascularity with a haphazard pattern and distortion of normal vascularity were observed. This haphazard vascularity is described as “crisscross” vessels. In sex cord stromal tumors, early enhancement followed by persistence of enhancement longer than normal testicular enhancement was documented. Adenomatoid tumors are extratesticular benign lesions that show an early enhancement and early wash-out after the administration of microbubble contrast. Epidermoid cysts and hematomas are evident by a clear lack of enhancement after intravenous contrast injection. Intratesticular abscesses demonstrate a nonvascular core with a hyperenhancing thick irregular rim. Nonenhancement is also characteristic of testicular infarcts, although perilesional rim enhancement may be visualized [12].

Due to angiogenesis and increased endothelial permeability in malignant masses, increased echoes and enhancement within lesions are generally associated with malignancies [13].

In a retrospective study to analyze the diagnostic performance of CEUS for testicular neoplasms, the authors concluded that a more precise and convincing diagnosis can be made by utilizing CEUS in testicular space–occupying lesions, particularly when conventional ultrasound is unable to differentiate the type of lesion, and use of the former can guide management as well as avoid unnecessary testicular resections [14]. They concluded that CEUS had a high degree of sensitivity and specificity in distinguishing malignant from benign testicular lesions. A distinct advantage noted by the authors was that CEUS could detect the small low-velocity vessels within the tumor, which can be missed on color and power Doppler examinations.

Few studies have also concluded that CEUS is superior to CT and MRI using contrast in identifying small vessels [15, 16].

In a study of over hundred patients, the authors observed high level of accuracy in diagnosing small testicular malignancies using unenhanced combined with CEUS with a 95% confidence interval [17]. In CEUS studies, they observed rapid wash-in and rapid wash-out in majority of testicular malignancies and this being the best parameter in differentiating malignant from benign masses. It was therefore concluded that quantitative CEUS is a useful adjunct in the ultrasound evaluation of testicular lesions and thus aids in the management.

As documented in several previously reported studies and case reports [17–19] involving testicular malignancies, early wash-in and early wash-out of contrast were demonstrated in our case as well. The feature of twisted blood vessels previously reported in mixed germ cell tumors [19] was also observed in our case. We could observe peripheral rim enhancement in our case similar to pseudocapsule sign documented in previous study [19]. One study [20] concluded germ cell tumors can be accurately differentiated from nongerm cell tumors in CEUS with an accuracy of 92.3% based on presence or absence of necrosis, although combining ultrasound findings with patient age and tumor markers is always helpful.

In conclusion, the use of microbubble contrast ultrasound is complementary in the characterization of focal testicular lesions. It aids in the characterization of such lesions, particularly those labeled as indeterminate on conventional ultrasound. The efficacy and safety of CEUS are well established and should be advocated as an adjunct to conventional ultrasound for the evaluation and management of focal testicular lesions.

## 7. Patient Perspective

The patient maintains satisfaction and content with the treatment, including contrast ultrasound, which enabled prompt surgical intervention, pathology evaluation, and subsequent chemotherapy. He remained in good health throughout treatment and is currently in remission.

## Data Availability

The data that support the findings of this study are available on request from the corresponding author. The data are not publicly available due to privacy or ethical restrictions.
